# Temporal patterns of Deepwater Horizon impacts on the benthic infauna of the northern Gulf of Mexico continental slope

**DOI:** 10.1371/journal.pone.0179923

**Published:** 2017-06-22

**Authors:** Michael G. Reuscher, Jeffrey G. Baguley, Nathan Conrad-Forrest, Cynthia Cooksey, Jeffrey L. Hyland, Christopher Lewis, Paul A. Montagna, Robert W. Ricker, Melissa Rohal, Travis Washburn

**Affiliations:** 1 Harte Research Institute for Gulf of Mexico Studies, Texas A&M University-Corpus Christi, Corpus Christi, Texas, United States of America; 2 Department of Biology, University of Nevada-Reno, Reno, Nevada, United States of America; 3 National Marine Fisheries Service, National Oceanic and Atmospheric Administration, Charleston, South Carolina, United States of America; 4 National Centers for Coastal Ocean Science, National Oceanic and Atmospheric Administration, Charleston, South Carolina, United States of America; 5 Industrial Economics, Incorporated, Cambridge, Massachusetts, United States of America; 6 Office of Response and Restoration, Assessment and Restoration Division, National Oceanic and Atmospheric Administration, Santa Rosa, California, United States of America; University of Illinois at Chicago, UNITED STATES

## Abstract

The Deepwater Horizon oil spill occurred in spring and summer 2010 in the northern Gulf of Mexico. Research cruises in 2010 (approximately 2–3 months after the well had been capped), 2011, and 2014 were conducted to determine the initial and subsequent effects of the oil spill on deep-sea soft-bottom infauna. A total of 34 stations were sampled from two zones: 20 stations in the “impact” zone versus 14 stations in the “non-impact” zone. Chemical contaminants were significantly different between the two zones. Polycyclic aromatic hydrocarbons averaged 218 ppb in the impact zone compared to 14 ppb in the non-impact zone. Total petroleum hydrocarbons averaged 1166 ppm in the impact zone compared to 102 ppm in the non-impact zone. While there was no difference between zones for meiofauna and macrofauna abundance, community diversity was significantly lower in the impact zone. Meiofauna taxa richness over the three sampling periods averaged 8 taxa/sample in the impact zone, compared to 10 taxa/sample in the non-impact zone; and macrofauna richness averaged 25 taxa/sample in the impact zone compared to 30 taxa/sample in the non-impact zone. Oil originating from the Deepwater Horizon oil spill reached the seafloor and had a persistent negative impact on diversity of soft-bottom, deep-sea benthic communities. While there are signs of recovery for some benthic community variables, full recovery has not yet occurred four years after the spill.

## Introduction

The deep sea is considered a generally stable environment where living organisms are less frequently challenged with steep changes in physical and chemical conditions compared to their coastal counterparts. Nevertheless, many different forms of natural disturbances that may disrupt the apparent ecosystem equilibrium of the deep sea have been observed, including benthic storms [1], mud slides [2], whale falls [3], and mass deposition of phytodetritus [4], among others. Subsequent recolonization and faunal successions of the affected areas are very slow in the deep sea [5]. The patchy nature of the deep-sea benthos may be a direct consequence of these disturbances because every “patch” may represent a community in a different faunal successional stage [6].

Seasonal and interannual variation of particulate organic matter (POM) transport to the deep sea through benthic-pelagic coupling is well documented [4,7]. Detritus derived from phytoplankton blooms may sink rapidly to the seafloor [8] and trigger a response by the benthic communities within a few days [9]. Within the northern Gulf of Mexico, phytoplankton blooms are commonly observed in the spring. These blooms are caused by the nutrient flux from the Mississippi and Atchafalaya Rivers, which peaks in the spring [10]. The abundance of benthic polychaetes on the northern Gulf of Mexico continental slope doubled in spring, compared to fall, most likely as a response to the increased availability of food [11]. Understanding seasonal and inter-annual dynamics is critical in order to separate community structure fluctuations in the natural background at non-impacted stations vs. post-spill successional processes at impacted stations.

In recent decades, deep-sea ecosystems have been experiencing ever-increasing pressures of newly introduced, anthropogenic disturbances. These include climate change and its influence on global ocean currents [12], destructive fishing techniques [13], mining of deep-sea minerals [14], and offshore oil and gas production [15]. The 2010 Deepwater Horizon (DWH) disaster in the Gulf of Mexico was an unprecedented deep-water oil spill. While the Gulf of Mexico is well known for natural hydrocarbon seeps along its continental margin, which harbor hydrocarbon oxidizing microbes and metazoan benthic organisms with special evolutionary adaptations to hydrocarbons, the DWH spill far exceeded all known natural seeps in the sheer amount of released hydrocarbons. In fact, the uncontrolled release of an estimated 3.19 million barrels of oil released from the Macondo oil well [16] during the 87 days following the blowout surpassed the estimated annual discharge of oil from all natural seeps in the entire Gulf of Mexico combined [17] by an order of magnitude, and in a concentrated area. In addition, nearly three million liters of dispersants with largely unknown environmental effects were released during the spill [18].

The DWH oil spill provides a unique opportunity to study the effects of hydrocarbon pollution on the deep-sea ecosystem over time. Our assessment is focused on responses of meiofauna and macrofauna communities of the soft-sediment benthos. In 2010, diversity and taxa richness of meiofauna and macrofauna were significantly depressed within an area of 172 km^2^ as a consequence of the hydrocarbon pollution [19]. Severe impacts of the oil spill on the benthos persisted in 2011 [20]. In the 172-km^2^ impact zone, contaminants remained high, macrofauna diversity and richness, and meiofauna richness remained impaired. Significantly higher macrofauna abundance and meiofauna diversity and a significantly lower nematode-to-copepod ratio in the impact zone in 2011, compared to 2010, were either signs of some recovery, or natural seasonal, or year-to-year variability. Multivariate analyses corroborated the persistent impacts of the DWH oil spill on the benthic communities in 2011. These analyses also indicated that a faunal succession had happened between 2010 and 2011 as analyses of similarity (ANOSIM) showed significant temporal differences for both meiofauna and macrofauna communities.

The overarching goal in the present study was to assess if meiofauna and macrofauna communities exhibited any evidence of recovery four years after the DWH oil spill. For this purpose, we analyzed abundance, richness, and diversity of meiofauna and macrofauna communities at 34 stations that were resampled in 2014 and compared the results to the 2010 and 2011 sampling periods. We also performed multivariate analyses to determine if benthic community succession occurred among the three sampling periods.

## Materials and methods

The impact of the oil spill on soft-bottom benthic communities was assessed on three different occasions: from 16 September through 30 October 2010 (two to three months after the well had been capped), from 23 May through 11 June 2011 (about eight months after the first sampling events), and from 29 May through 28 June 2014 (almost four years after the well had been capped). The 2010 cruises were conducted aboard the R/V *Gyre* and R/V *Ocean Veritas*, the 2011 cruise was conducted aboard the M/V *Sarah Bordelon*, and the 2014 cruise was conducted aboard the M/V *Irish*. A Bowers and Connelly multiple corer, the Mega Corer model, manufactured by OSIL (http://www.osil.co.uk/), was used to collect 12 sediment-core samples with each deployment. Three cores from each drop were used for macrofauna analysis and one was used for meiofauna analysis. The remaining cores were reserved for analyses of hydrocarbons, heavy metals, pore-water chemistry (Eh, sulfides, ammonia), and other sediment properties (total carbon, total organic carbon, total inorganic carbon, total nitrogen, grain size). The cores had an inner diameter of 10 cm. Meiofauna cores were subsampled with a smaller core of 5.5 cm inner diameter. Macrofauna cores were separated into 0–5 cm and 5–10 cm sediment sections in the 2010 samples and into 0–3 cm, 3–5 cm, and 5–10 cm sections in the 2011 and 2014 samples. Meiofauna cores were divided into 0–1 cm and 1–3 cm sediment sections. The different sections from each core were processed and analyzed for meiofauna and macrofauna abundance separately.

**Fig 1:**
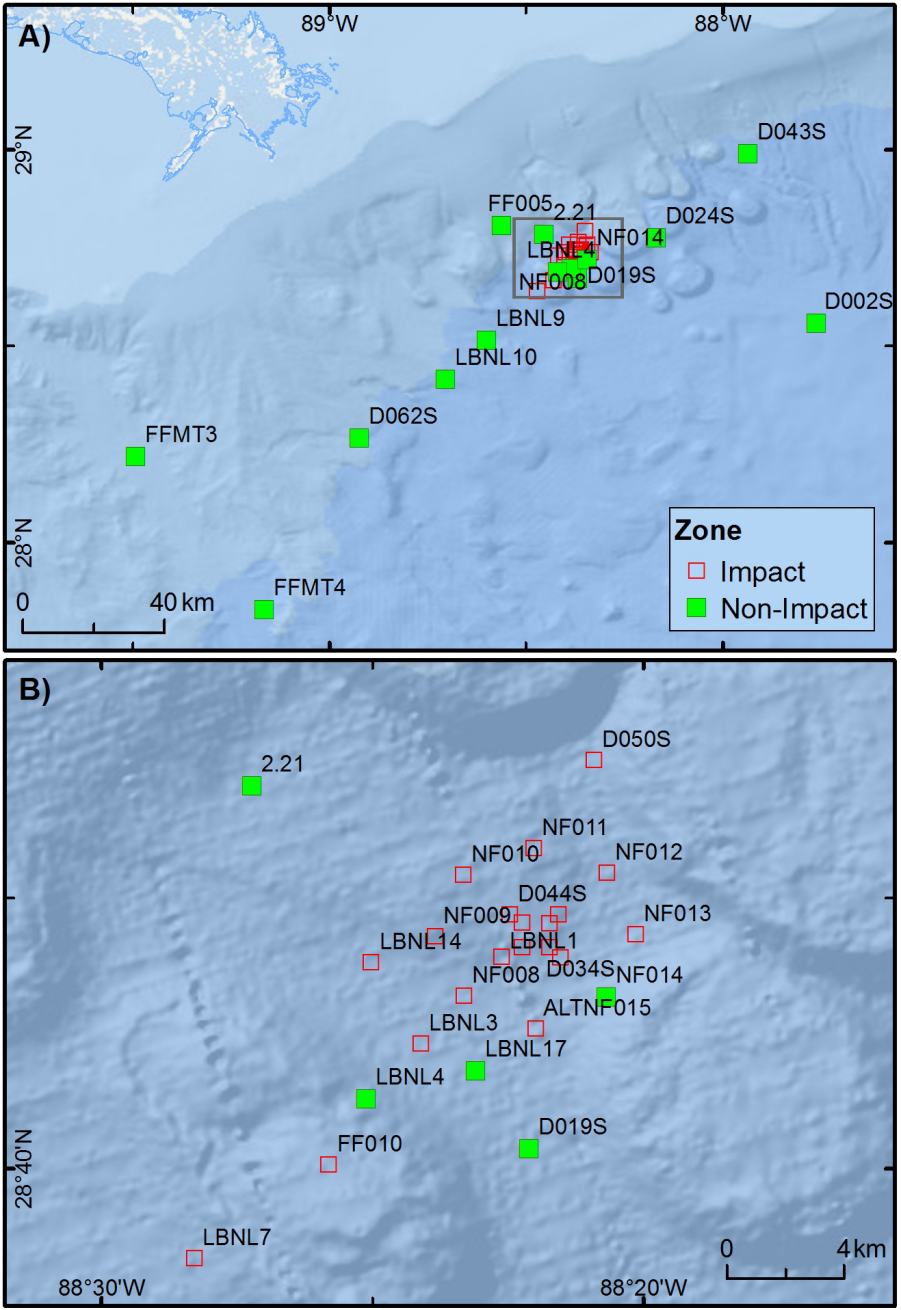
Station locations, representing impact zone (open red squares) and non-impact zone (solid green square), as defined by [19]. (A) All stations within impact and reference zones. (B) Zoomed to within 10 km from the MC252 wellhead.

The experimental approach was to test for significant differences in the concentration of chemical compounds related to oil spills and faunal community metrics between impact and non-impact zones and between the three sampling years (2010, 2011 and 2014). A total of 34 stations were sampled (Fig 1A and 1B) and classified as either “impact station” or “non-impact station”, depending in which zone, as defined by [19], it was located in. Stations located in high impact and moderate impact zones (sensu [19]) were classified as “impact zone”; stations located in areas with uncertain impacts and unlikely impacts (sensu [19]) were classified as “non-impact zone”. The establishment of these zones by [19] was based on a principal component analysis, which included environmental and biotic variables. Of the 34 stations 20 were in the impact zone (ALTNF001, ALTNF015, D031S, D034S, D040S, D042S, D044S, D050S, FF010, LBNL1, LBNL3, LBNL7, LBNL14, NF006MOD, NF008, NF009, NF010, NF011, NF012, and NF013) and 14 were in the non-impact zone (2.21, D002S, D019S, D024S, D043S, D062S, FF005, FFMT3, FFMT4, LBNL4, LBNL9, LBNL10, LBNL17, and NF014).

Stations were thus nested within the zones (“impact” or “non-impact”). The experimental design is a partially hierarchical, 2-way ANOVA (analysis of variance) that can be described by the following statistical model: Y_ijkl_ = μ + α_j_ +β_k_ + αβ_jk_ +γ_k(l)_ + αβγ_jk(l)_ +℮_(i)jkl_ where Y_ijkl_ is the dependent response variable; μ is the overall sample mean; α_j_ is the main fixed effect for year where *j* = 1, 2, or 3 for either 2010, 2011 or 2014; β_k_ is the main fixed effect for sampling zone where *k* = 1 or 2 for either the impact or non-impact zone; αβ_jk_ is the main fixed effect for the interaction between year and zone; γ_k(l)_ is the main effect for stations that are nested (or unique) to the zones and are thus a random effect as denoted by the parentheses around the subscript *l* that represents the 34 stations all of which are nested unique to one of the two zones; αβγ_jk(l)_ is the interaction term for year, zone, and station; and ℮_(i)jkl_ is the random error term for each of the *i* replicate measurements.

Because of sampling constraints and our rationale that meiofauna were assumed to vary little on the spatial scales, only one core sample was collected for meiofauna analysis from each single multi-corer drop at each station. Because there are no replicate cores within multi-corer drops, the triple-interaction term does not exist, thus the model is reduced to: Y_ijk_ = μ + α_j_ +β_k_ + αβ_jk_ + γ_k(l)_ +℮_(i)jkl_. All analysis-of-variance (ANOVA) tests were performed using SAS 9.4 software, as described in [19].

The most abundant macrofauna taxa, Polychaeta, Mollusca, and Crustacea (Malacostraca), were identified to family level. The remaining macrofauna organisms were identified to order (e.g. Podocopida) or higher taxonomic levels (e.g. Nemertea, Sipuncula). Meiofauna were identified to order level (e.g. Amphipoda, Isopoda) or higher (e.g. Nematoda, Copepoda). Macrofauna and meiofauna community structure was analyzed by creating a Bray-Curtis similarity matrix among stations using abundance data from taxa listed above and then plotting the results in a non-metric multi-dimensional scaling (MDS) ordination [21,22]. Differences in community structure between years and zones were tested using ANOSIM and SIMPER in Primer [21]. One-way ANOSIM analyses were conducted to test for significant differences between impact and non-impact zone in 2014, as well as significant temporal differences between benthic communities in 2010, 2011, and 2014. Abundance data were fourth-root transformed prior to multivariate analysis in Primer to decrease the effect of numerically dominant species [23].

Benthic response variables presented here include total faunal abundance, richness (number of taxa per sample), diversity (Hill’s N1) [24], and nematode-to-copepod ratios (N:C). Hill’s N1 is the exponentiated form of the Shannon-Wiener H' diversity index, N1 = ℮^H'^ and was selected because it is easily interpreted as the effective number of dominant taxa and trends to 1 as diversity decreases. The N:C ratio was used because it is a robust indicator for environmental disturbances, including hydrocarbon exposure [15,19]. Abiotic environmental variables consisted of total petroleum hydrocarbons (TPH), total polycyclic aromatic hydrocarbons (total PAHs), barium, and selected natural habitat characteristics (depth, sediment grain size, and total organic carbon (TOC).

No specific permissions were required to sample any of the locations reported on in this study. Field studies did not involve endangered or protected species.

Data sets of environmental variables, macrofauna, and meiofauna were made publicly available on the Gulf of Mexico Research Initiative Information and Data Cooperative (GRIIDC), where they were assigned the unique dataset identifier (UDI) R4.x267.000:0018, R4.x267.000:0019, and R4.x267.000:0020, respectively.

## Results

### Taxonomic composition

Polychaetes dominated the macrofauna assemblages. In the impact zone they accounted for 82.6% (± 5.7% SD) in 2010 and 82.9% (± 8.1%) in 2011, while their relative numerical dominance in the non-impact zone was less pronounced with 70.5% (± 7.1%) in 2010 and 70.0% (± 5.9%). In 2014 the relative contribution of polychaetes decreased. However, their relative abundance of 74.4% (± 6.3%) in the impact zone still exceeded the non-impact zone (63.5 ± 7.6%). In the non-impact zone the most abundant polychaete families were Maldanidae, Paraonidae, Spionidae, and Capitellidae. Their relative numbers and abundance ranks slightly varied between the three sampling years, but they each consistently accounted for 5.3%-11.5% of the average total macrofauna counts. In the impact zone fluctuations of different polychaete families was much more pronounced between sampling years. Furthermore, dominance of the most abundant families in the impact zone was more eminent, i.e. their relative abundances attained higher values than in the non-impact zone. In 2010 the most abundant families were Dorvilleidae (20.4 ± 19.6%), Paraonidae (14.2 ± 5.8%), Maldanidae (11.6 ±2.5%), Capitellidae (9.8 ± 3.7%), and Spionidae (6.3 ± 2.6%). The high standard deviation of the relative dorvilleid abundance indicates the large spread between some moderately impacted stations where Dorvilleidae accounted for less than 5% and heavily impacted stations where they accounted for up to 61.1%. In 2011 the dominance of dorvilleid polychaetes had further increased, as they accounted for 26.0% (± 28.4%) of the macrofauna counts. The large standard deviation is once again explained by the large spread of relative abundances across the different stations. At four of the heavily impacted stations within about 1 km of the well, dorvilleids accounted for more than 70% of the macrofauna, whereas in some of the moderately impacted stations their counts were very low and would account for less than 5%. The other polychaete families that accounted for at least 5% of the total macrofauna count in 2011 were Maldanidae (10.2 ± 2.0%), Paraonidae (9.7 ± 5.5%), Capitellidae (7.4 ± 4.0%), and Acrocirridae (5.0 ± 3.3%). In 2014 numbers of Dorvilleidae had plummeted and accounted for only 2.6% (± 4.0%) of the macrofauna, with a maximum of 13.8% at one of the heavily affected stations. The most abundant polychaete families in the 2014 impact zone were Cirratulidae (12.0 ± 8.1%), Paraonidae (9.9 ± 5.1%), Capitellidae (8.2 ± 4.1%), Syllidae (6.4 ± 3.9%), Maldanidae (6.3 ± 1.5%), and Spionidae (6.2 ± 2.7).

Mollusks had higher absolute and relative abundances in the non-impact zone, compared to the impact zone, in 2010 and 2011. In the non-impact zone they accounted for 11.8% (± 6.8%) in 2010 and 10.5% (± 4.5%) in 2011. In the impact zone they accounted for 7.1% (± 3.3%) and 6.8% (± 3.8%), respectively. In 2014 the relative abundance of mollusks increased in the non-impact zone to 12.8% (± 2.7%). In the impact zone the relative contribution of mollusks nearly doubled in 2014 to 13.2% (±4.8%). This strong increase in the 2014 impact zone was mostly caused by the bivalve family Thyasiridae, which accounted for 9.1% (± 4.9%) of the total macrofauna count. In 2010 and 2011 thyasirid bivalves contributed only 2.3% (± 2.1%) and 1.8% (± 1.8%), respectively. Thyasiridae also increased in the non-impact zone to 6.1% (± 3.5%), compared to 2.0% (± 2.6%) in 2010 and 3.2% (± 3.7%) in 2011. The second most abundant mollusk taxon was the aplacophoran family Prochaetodermatidae, which was consistently more frequent in the non-impact zone (2.5 ± 2.7% to 3.4 ± 4.7%) throughout the three sampling periods, compared to the impact zone (0.7 ± 0.9% to 1.6 ± 1.2%).

Crustaceans were considerably more abundant in the non-impact zone, compared to the impact zone, throughout all three sampling years. In the non-impact zone they accounted for 11.8% (± 6.8%) in 2010, 11.6% (± 4.5%) in 2011, and 15.8% (± 6.1%) in 2014. In the impact zone their relative contributions to the macrofauna total were 3.2% (± 2.6%) in 2010, 2.8% (± 2.6%) in 2011, and 5.9% (± 3.7%) in 2014. We identified almost 60 families and six orders of crustaceans. Most of the crustacean taxa were rare. The most abundant crustaceans were ostracods of the order Podocopida and the tanaid family Colletteidae., both of which peaked in the 2014 non-impact zone contributing 3.5% (± 2.3%) and 1.8% (± 1.6%) to the macrofauna counts, respectively. Among the remaining taxa, which were identified to order, class, or phylum level, only Nemertea were consistently abundant. Their relative contribution varied relatively little between zones or sampling years, ranging from 3.0% (± 2.0%) to 4.9% (± 2.5%).

The two dominant meiofauna taxa were Nematoda and Harpacticoida. These two groups combined accounted for more than 90% at any station and sampling time. When station abundances were averaged within each sampling year and zone, nematodes and harpacticoids together contributed between 97.3% (± 2.0%) (in the 2010 non-impact zone) and 99.4% (± 0.75%) (in the 2010 impact zone) to the total meiofauna counts. Nematoda were always the most abundant meiofauna taxon, though their absolute and relative contribution varied between impact and non-impact zones and between sampling years. Nematodes reached their highest abundance in the impact zone in 2010 when they averaged 3,334 n/10 cm^2^ and accounted for 92.2% (± 5.8%) of the meiofauna community. Nematode numbers in the impact zone declined in 2011 and 2014 to 2,085 n/10 cm^2^ and 1,757 n/10 cm^2^, respectively. In the non-impact zone, nematode numbers were quite consistent over the years (1,654 n/10 cm^2^ to 1,726 n/10 cm^2^). Copepoda abundance was lowest in the 2010 impact zone, averaging 195 n/10 cm^2^, which accounted for only 6.9% (± 5.2%) of the overall meiofauna community. Copepods increased in the impact zone in 2011 to 348 n/m^2^ (15.8 ± 9.4%), but declined in 2014 to 238 n/m^2^ (10.8 ± 6.4%). In the non-impact zone, copepod abundance steadily decreased from 2010 (417 n/10 cm^2^, 19.5 ± 10.9%) to 2011 (262 n/10 cm^2^, 12.8 ± 5.4%) and to 2014 (197 n/10 cm^2^, 10.4 ± 3.2%). Other meiofauna taxa that were represented in the samples were (in order of their overall abundance): Polychaeta, Ostracoda, Kinorhyncha, Bivalvia, Tardigrada, Aplacophora, Tanaidacea, Loricifera, Amphipoda, Sipuncula, Acari, Isopoda, Gastrotricha, Cnidaria, Gastropoda, Turbellaria, Nemertea, Rotifera, Oligochaeta, Priapulida, and Echinodermata. The contribution of any of these taxa was relatively minor.

### Time series

**Fig 2:**
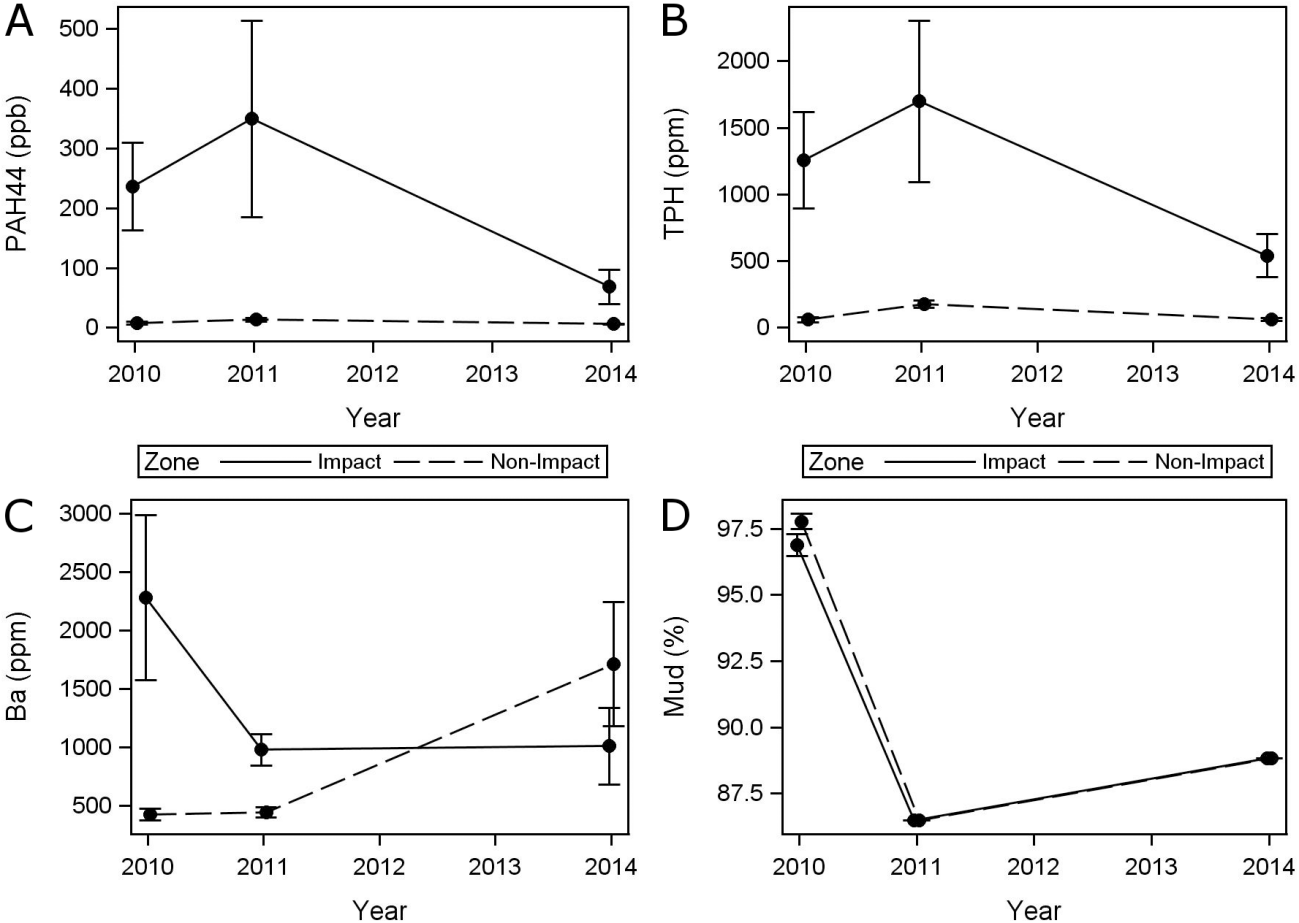
Temporal patterns of sediment chemistry in impact and non-impact zones, including mean and standard errors. **(**A) Total Polycyclic Aromatic Hydrocarbons for 44 priority compounds (PAH44). (B) Total Petroleum Hydrocarbons (TPH). (C) Barium (Ba). (D) Mud, i.e., total silt plus clay content.

The chemical-contaminant indicators of the oil spill, PAH44 and TPH, were consistently higher in the impact zone than non-impact zone (Tables 1 and 2, Fig 2A and 2B). TPH was 1038% higher and PAH44 was 224% higher. In contrast, the mud (total silt plus clay) content was the same (91%) in both zones (Fig 2D). The means of PAH44 and TPH appear to change over time (Fig 2A and 2B). However, these changes were not statistically significant as the Year*Zone interaction terms are non-significant (at P < 0.05) with P values of 0.2353 for PAH44 and 0.1604 for TPH (Table 2).

**Table 1:** Means and standard errors (SE) for chemical and faunal differences between impact distances, averaged across all sampling years.

Group	Variable (units)	Impact		Non-Impact	
Chemistry	PAH (ppb)	218.2	(61.6)	13.9	(9.3)
	TPH (ppm)	1165.9	(244.8)	102.4	(13.9)
	Ba (ppm)	1425.4	(270.7)	862.1	(198.4)
	Mud (%)	90.7	(0.6)	91.0	(0.8)
Macrofauna	Abundance (n/m^2^)	10,229	(450)	9,325	(383)
	Richness (taxa/sample)	24.80	(0.61)	29.93	(0.68)
	Diversity (N1/sample)	16.40	(0.53)	21.74	(0.48)
Meiofauna	Abundance (n/10 cm^2^)	2,677	(192)	1,984	(171)
	Richness (taxa/sample)	7.85	(0.30)	10.19	(0.38)
	Diversity (N1/sample)	1.51	(0.03)	1.69	(0.04)
	N:C	15.84	(2.57)	7.41	(0.58)

**Table 2:** Analyses of variance (ANOVA) probabilities (P values) for chemical and faunal differences between years and impact zones.

Group	Variable (units)	Year	Zone	Year*Zone
Chemistry	Total PAHs (ppb)	0.2033	**0.0249**	0.2353
	TPH (ppm)	0.0763	**0.0114**	0.1604
	Ba (ppm)	0.2379	0.1056	**0.0174**
	Mud (%)	**<0.0001**	0.1118	0.0767
Macrofauna	Abundance (n/m^2^)	**0.0006**	0.3659	**0.0076**
	Abundance (Ln(n+1)/m^2^)	**0.0027**	0.6472	**0.0150**
	Richness (taxa/sample)	**<0.0001**	**0.0249**	0.3009
	Diversity (N1/sample)	**<0.0001**	**0.0058**	0.1229
Meiofauna	Abundance (n/10 cm^2^)	**0.0003**	0.0576	**0.0499**
	Abundance (Ln(n+1)/10 cm^2^)	**0.0104**	0.1263	0.0962
	Richness (taxa/sample)	**<0.0001**	**0.0002**	0.2004
	Diversity (N1/sample)	0.1721	**0.0002**	**<0.0001**
	N:C	**0.0285**	**0.0047**	**0.0013**

Significant differences (P < 0.05) in bold font.

Barium (Ba) concentration did change between years and zones because the interaction was significant (Table 2, P = 0.0174). Ba concentrations in the non-impact zone were similar in 2010 and 2011. However, average Ba concentrations increased from 2011 to 2014. This increase was due to an unexplained change at three stations. From 2011 to 2014, Ba increased from 336 to 6,963 ppm at station 2.21, from 255 to 6550 at station D031S, and from 316 to 1650 at station D043S.

**Fig 3:**
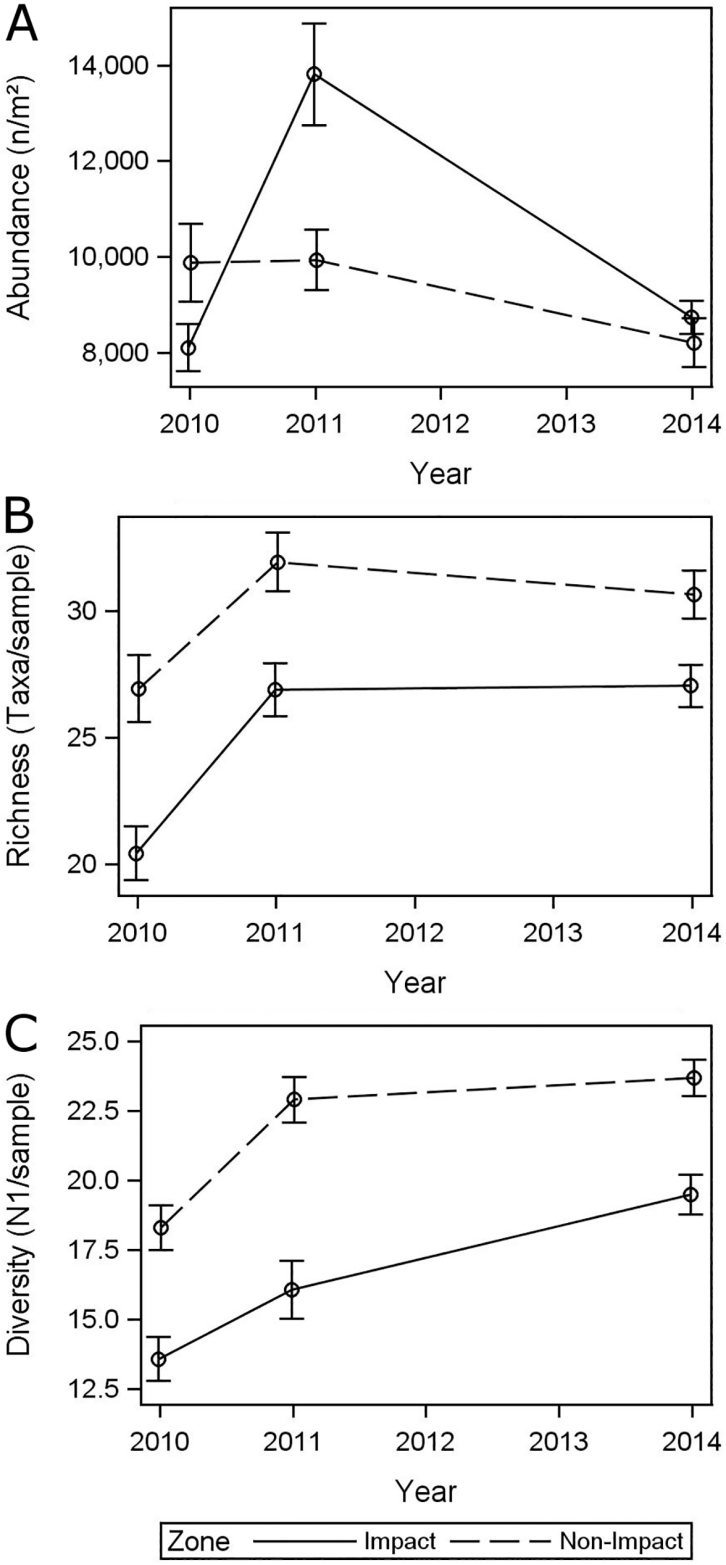
Temporal patterns of macrofauna metrics in impact and non-impact zones, including mean and standard errors. (A) Abundance (n/m^2^). (B) Taxa richness (S, number of taxa). (C) Diversity (Hill’s N1).

Following a sharp increase in abundance by 69% between 2010 and 2011 in the impact zone, macrofauna abundance decreased between 2011 and 2014 by approximately 37% (Fig 3A). In the non-impact zone macrofauna abundance decreased by 17% between 2011 and 2014. Nevertheless, average abundance in the impact zone was still slightly higher (8,733 n/m^2^) than in the non-impact zone (8,208 n/m^2^) in 2014. The significant time-zone interaction term (P = 0.0076) (Table 2) indicates that changes in abundance between 2010 and 2014 were significantly different between impact and non-impact zones over the different time periods. By 2014, the abundance patterns for the two zones converged with overlapping 95% confidence intervals. Macrofauna richness and diversity in the impact zone continued to be significantly lower than in the non-impact zone (Table 2). The metrics were approximately 21% and 13% lower for richness and diversity, respectively (Fig 3B and 3C). While diversity steadily increased in the impact zone between 2010 and 2014, richness increased between 2010 and 2011, but remained virtually the same between 2011 and 2014. The non-impact zone experienced only subtle changes in these metrics between 2011 and 2014 as the number of taxa slightly decreased, but diversity slightly increased. The non-significant time-zone interaction term for richness and Hill’s N1 diversity suggests that both metrics have not yet converged within the four years following the oil spill, although the persistent increases in diversity within the impact zone from 2010 to 2014 suggest a possible trend toward recovery for this variable.

**Fig 4:**
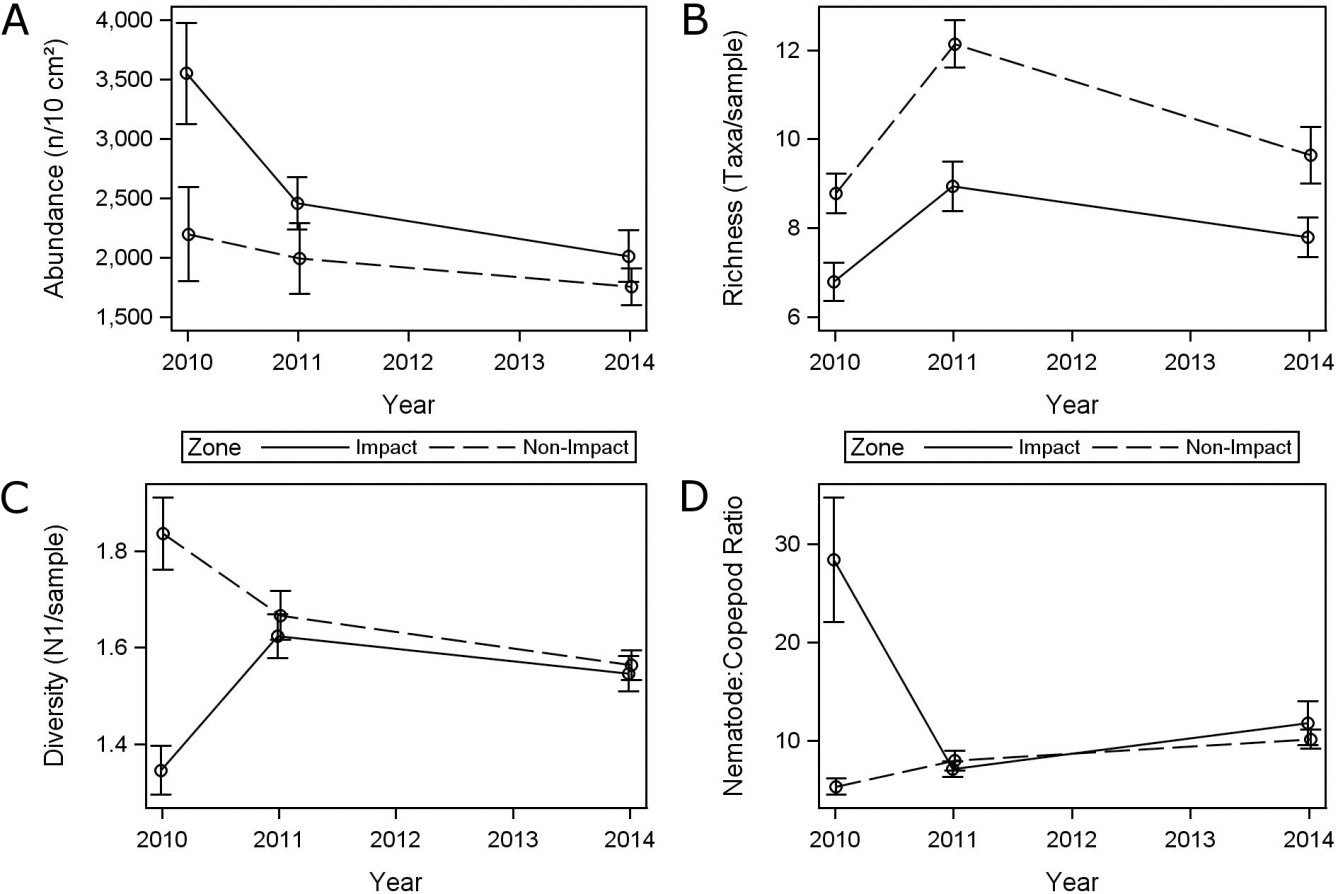
Temporal pattern of meiofauna metrics in impact and non-impact zones, including mean and standard errors. (A) Abundance (n/10 cm^2^). (B) Taxa richness (S, number of taxa). (C) Diversity (Hill’s N1). (D) Nematode:Copepod ratio.

There was little change in any of the meiofauna metrics between 2011 and 2014, in both impact and non-impact zones (Fig 4). Abundance levels of both zones became more similar, due to a stronger decrease in meiofauna numbers at impact stations (Fig 4A). Meiofauna abundance had decreased in the impact zone and in the non-impact zone by 17.5% and 5%, respectively since 2011. Absolute abundances averaged across impact stations were still higher (2,029 n/10 cm^2^) than at non-impact stations (1,891 n/10 cm^2^) in 2014. Meiofauna abundance was significantly different between the three sampling periods (P = 0.0003), but not between zones (P = 0.0576) (Table 2). Meiofauna diversity and nematode-to-copepod ratios in 2014 were similar to 2011 levels, in both impact and non-impact zones (Fig 4C and 4D). Diversity across all three sampling events was significantly higher in the non-impact zone (P = 0.0002). This was caused by differences in 2010 when 10.3 taxa were found. In 2011 (8.6 taxa) and 2014 (7.6 taxa), diversity was not significantly different (Tukey test). The nematode-to-copepod ratio was significantly different between impact and non-impact stations across the sampling events, i.e., the interaction between year and zone was significant (P = 0.0013) (Table 2). This was mainly caused by the much higher ratio at impact stations in 2010. In 2011 and 2014 differences between both zones were minor. Taxa richness was the only meiofauna metric that was consistently significantly different between impact and non-impact zones throughout the three sampling events (Fig 4B). Richness remained significantly higher in the non-impact zone (P = 0.0002), with non-significant interaction term between time and zone (P = 0.2004).

### Vertical distribution

**Fig 5:**
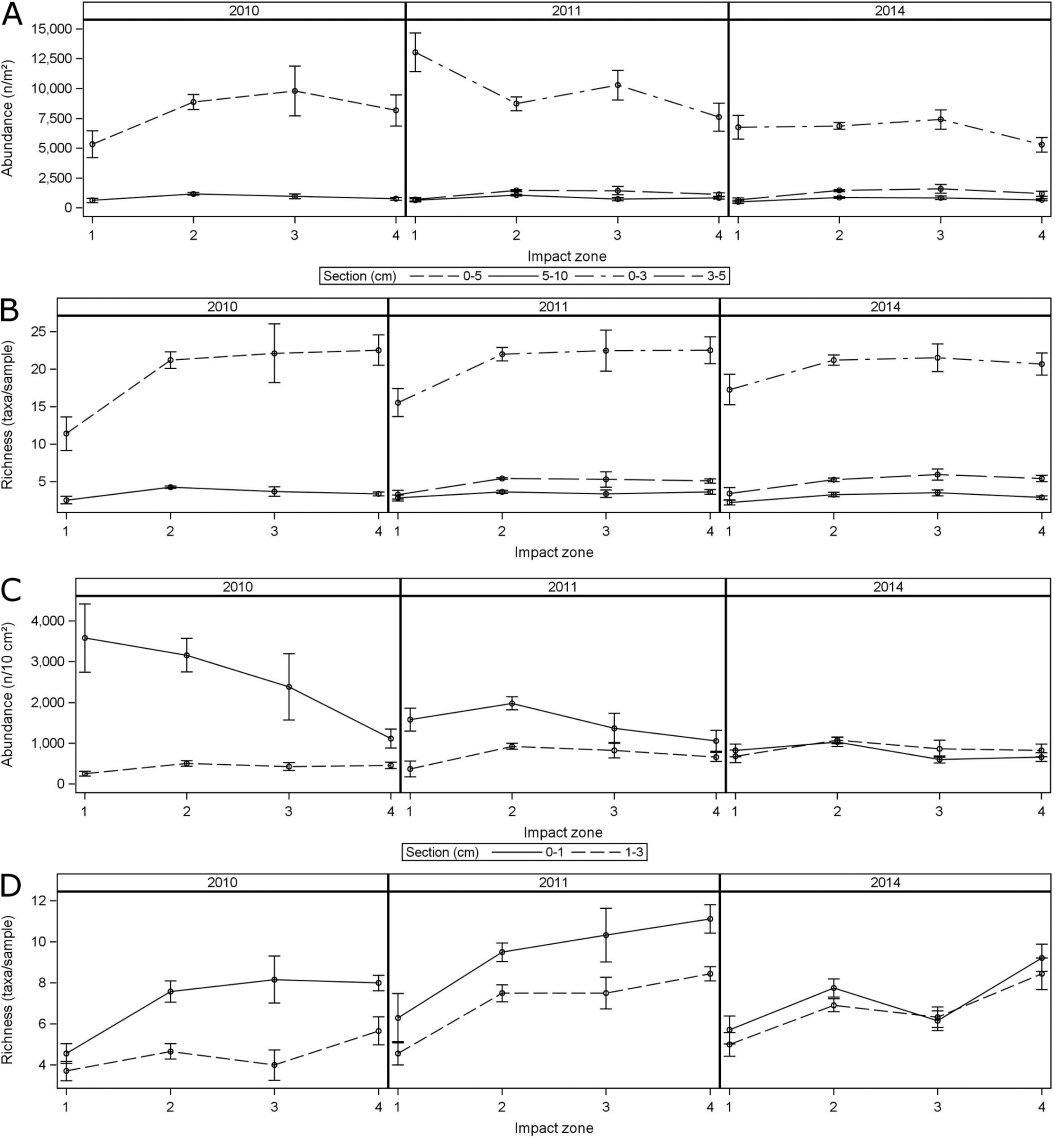
Interactions between vertical sediment sections, year, and zone treatments for benthic community metrics including mean and standard errors. Impact zones represent: 1. High impact; 2: Moderate impact; 3: Uncertain impact; 4: Unlikely impact, as defined by [19]. **(**A) Macrofauna abundance (n/m^2^). (B) Macrofauna taxa richness (S, number of taxa). (C) Meiofauna abundance (n/10 cm^2^). (D) Meiofauna taxa richness (S, number of taxa).

The vertical distribution of macrofauna and meiofauna was examined in the four zones independently rather than pooling zones (i.e., 1 and 2 into impact and 3 and 4 into non-impact). The zones were analyzed separately to test if there was an effect of distance from the wellhead rather than overall impact. Macrofauna abundance was highest in the top 0–5 cm (2010 samples) or 0–3 cm (2011 and 2014 samples) sections in all four impact zones (Fig 5A). The 5–10 cm (2010 samples) or 3–5 cm and 5–10 cm (2011 and 2014 samples) were only sparsely populated by macrofauna organisms throughout all impact zones and sampling events. The most distinct temporal variability in macrofauna abundance was found in the upper sediment layer of the highly impacted zone near the wellhead. In 2010 macrofauna abundance in this zone was significantly lower than in any other zone. In 2011 the highly impacted stations had significantly higher macrofauna abundance than the other zones, mainly caused by very high numbers of dorvilleid polychaetes. In 2014 macrofauna abundance was comparable to the moderately impacted and background stations. Macrofauna taxa richness was by far highest in the top sediment layers throughout all impact zones and sampling events (Fig 5B). In 2010 taxa richness in the high impact zone was significantly lower than in the moderately impacted and background stations. In 2011 taxa richness in the high impact zone had increases considerably, but was still significantly lower than in the three other zones. In 2014 the high impact zone’s taxa richness had further increased, even though it was still significantly lower than in the other zones.

Meiofauna abundance in 2010 was highest in the top 0–1 cm sediment section of the high impact zone (Fig 5C). It continually decreased from the high impact to the background zone. In the 1–3 cm sediment sections, meiofauna abundance was significantly lower in each of the four impact zones. In 2011 meiofauna abundance in the 0–1 cm sediment section had decreased significantly in impact zones one, two, and three, whereas the 1–3 cm section had increased in all four zones. In 2014 abundance in the top section continued to decrease in each of the four impact zones, whereas it increased in the 1–3 cm section of the high impact zone. Because of the consistent decrease in abundance in the 0–1 cm sediment section and the slight increase in the 1–3 cm sections between 2010 and 2014, meiofauna abundance was similar between the 0–1 cm and 1–3 cm sections in all four impact zones in 2014. Furthermore, meiofauna abundance was not significantly different between any of the four impact zones in 2014. Meiofauna taxa richness was significantly lower in the high impact zone in 2010 (Fig 5D). In 2011 taxa richness in the high impact zone had increased. However, moderate impact and background zones had also increased taxa richness in 2011. In 2014 taxa richness of the high impact zone stayed on a similar level, whereas the moderate and impact zones approximately returned to the lower levels of 2010. This means that meiofauna taxa richness was still lowest in the high impact zone, but that the values have been converging.

### Community structure

**Fig 6:**
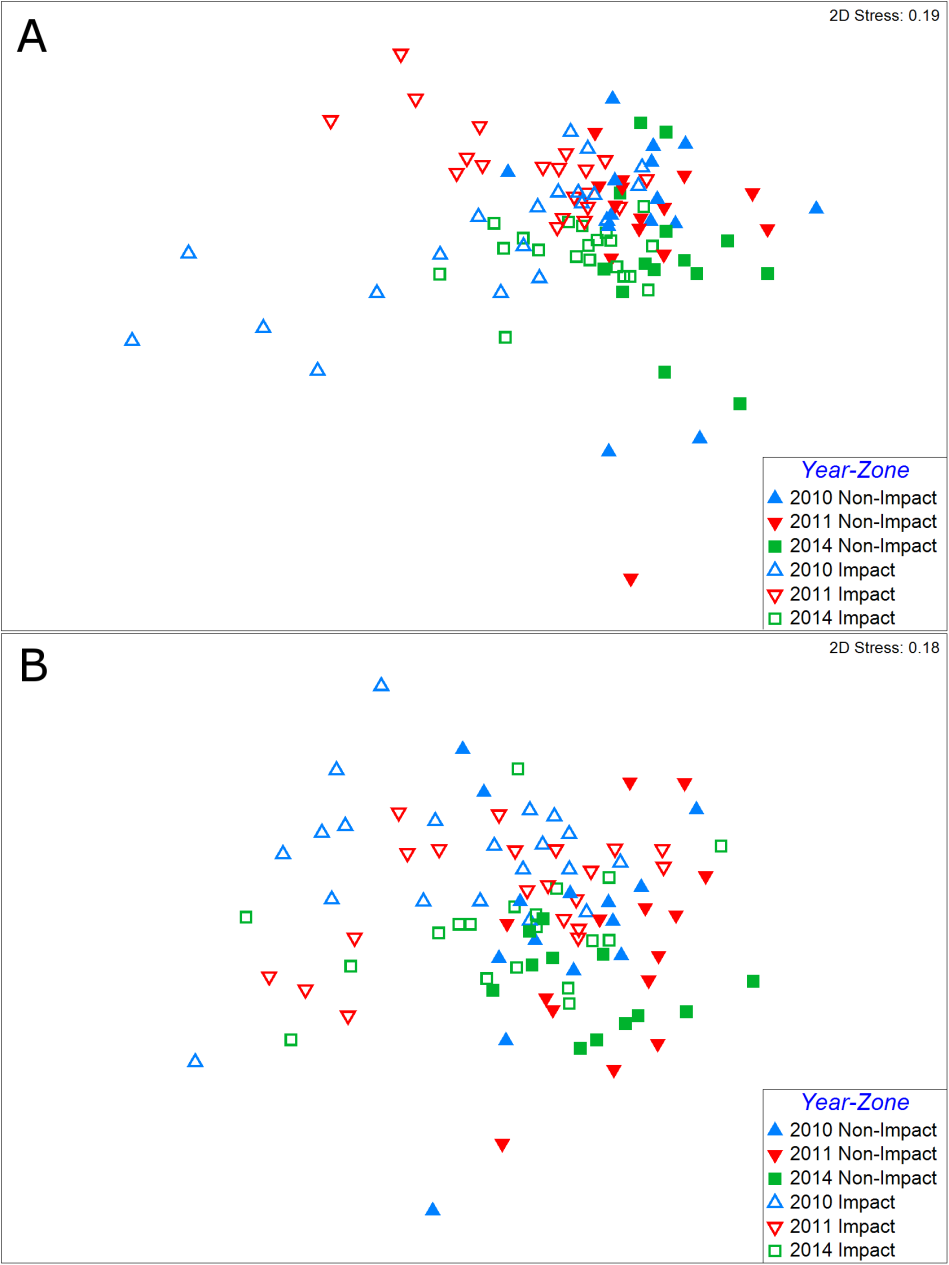
Non-metric multidimensional scaling plots of benthic community structure with respect to the 2-way design (year vs. zone). (A) Macrofauna. (B) Meiofauna.

ANOSIM analyses confirmed that significant differences in the macrofauna community structure between impact and non-impact zone, which had been observed in the 2010 and 2011 samples, continued to exist (P < 0.0003). Furthermore, impact zone communities showed distinct patterns of faunal succession stages with statistically significant differences in the benthic communities between 2010 and 2011 in 2014 (P = 0.0006), between 2010 and 2014 (P = 10^−5^), and between 2011 and 2014 (P = 10^−5^). Non-impact zone communities were not significantly different between 2010 and 2011 (P = 0.244). However, between 2010 and 2014 (P = 0.002) and 2011 and 2014 (P = 0.0008) they did change significantly. In the two-dimensional MDS plot (Fig 6A), impact-zone stations (open symbols) were consistently further to the left than non-impact stations (solid symbols), but a larger dispersion pattern is noticed in the 2010-impact zone samples. While throughout different sampling years (different colors) macrofauna at non-impact stations experienced only subtle changes, a temporal pattern for communities at impact stations is evident: the 2010 samples (blue symbols) were located in the median left part of the MDS plot, the 2011 samples (red symbols) in the upper left, and the 2014 samples (green symbols) did not extend to the far left, but were more centered.

Meiofauna communities in 2014 were not significantly different between impact and non-impact zones (ANOSIM: P = 0.116). However, there was a significant change in the impact zone meiofauna communities over time, as significant differences were found between 2010 and 2011 (P = 0.014), between 2010 and 2014 (P = 0.002), and between 2011 and 2014 (P = 0.021). The two dominant taxa, which contributed most to the community turnover, were nematodes and copepods. Nematodes were very abundant in the impact zone in 2010 (3,334 n/10 cm^2^), causing the high overall meiofauna abundance and the high nematode-to-copepod ratio. Nematode numbers had strongly declined in 2011 (2,085 n/10 cm^2^) and had continued to decline by 2014 (1,757 n/10 cm^2^). In contrast, copepods had strongly increased between 2010 (195 n/10 cm^2^) and 2011 (348 n/10 cm^2^), but then decreased again by 2014 (238 n/10 cm^2^). The non-impact stations also changed significantly between 2010 and 2011 (P = 0.039), between 2010 and 2014 (P = 4 x 10^−5^), and between 2011 and 2014 (P = 0.002). In the MDS analysis (Fig 6B), impact stations tended to be to the left side of the plot. Compared to 2010 and 2011, only a few of the impact stations were located to the far left of the plot, whereas most of them were closer to the non-impact stations near the center. Similarly, data in Fig 4 show that trends over time for some meiofauna variables (abundance, Hill’s N1 diversity, and N:C ratios) within the impact versus non-impact zones have converged by 2014.

## Discussion

### Persistence of impacts

Benthic macrofauna communities remained impaired four years after the DWH blowout as macrofauna richness and diversity were still significantly lower in the impact zone in contrast to the non-impact zone. The 69% increase in macrofauna abundance in the impact zone in 2011 [20], as well as the decline in 2014 was primarily caused by fluctuations of the polychaete families Dorvilleidae and, to a lesser degree, Acrocirridae. In 2011 Dorvilleidae was the dominant taxon in the impact zone, accounting for an average of approximately 26% of all macrofauna and up to 80% at stations particularly close (~1 km) to the wellhead. The average Dorvilleidae density of 4,802 n/m^2^ across all impact-zone stations in 2011, had declined by 95% in 2014 to an average density of 205 n/m^2^. Acrocirridae had declined by 69% between 2011 and 2014, from 803 n/m^2^ to 249 n/m^2^. The decline of these dominant taxa increased the evenness of overall population distributions among taxa, which ultimately caused the increase of the diversity metric in 2014. Causes for these fluctuations in abundance are unknown. Seasonal variations can be excluded because the 2011 and 2014 samples were all sampled in May and June, but inter-annual variability is possible. The multivariate analyses confirmed that the macrofauna in the impact zone remained significantly different from the non-impact zone. The MDS plot shows a trajectory for impact-zone stations between 2010 and 2014, which suggests that notable changes in the communities have occurred between each sampling event. Overall, patterns in the MDS plot indicate that the macrofauna communities within the impact zone were more similar to the non-impact zone communities in 2014, in comparison to the previous sampling events. Particularly, the most contaminated stations near the wellhead did not extend as far to the left in the MDS plot in2014, as they did in 2010 and 2011. These stations seem to have recovered as they were less dominated by dorvilleid polychaetes and other opportunistic taxa. This conclusion is corroborated by the increasing taxa richness in the high impact zone in the near-surface sediment layers.

Trends over time for some meiofauna variables (abundance, Hill’s N1 diversity, and N:C ratios) within the impact versus non-impact zones converged by 2014, which may indicate signs of recovery. However, meiofauna communities had not fully recovered from the hydrocarbon contamination four years after the blowout because the taxonomic richness in the impact zone remained significantly lower. The non-significant interaction term between time and zone means that the slight increase in richness between 2010 and 2011 and the slight decrease between 2011 and 2014 were approximately parallel in both impact and non-impact zones. This indicates that natural fluctuations occurred at both impact and non-impact stations. Similar levels of abundance, diversity, and N:C ratios in impact and non-impact zones in 2014 indicate that meiofauna communities showed some signs of recovery from the hydrocarbon contamination. The N:C ratio is a good indicator of pollution by petroleum hydrocarbons [25] so its convergence between impact and non-impact zones in 2011 and 2014 is an indicator of recovery. The conclusion that some meiofauna recovery has occurred is corroborated by the results of our multivariate analysis, which found no significant difference between meiofauna communities of impact and non-impact zones in 2014. Three stations that were within approximately 1 km of the oil well were still located to the far left of the MDS plot in 2014. These stations are most likely still heavily affected by the oil contamination, whereas most other stations seem to show more signs of recovery. The fact that meiofauna communities of non-impact stations had significantly changed between 2010 and 2011 and between 2011 and 2014 indicates that they undergo natural patterns of temporal dynamics. This further implies that the significant temporal changes in meiofauna communities observed within the impact zone may be the result of processes related to both recovery from the DWH contamination and natural community dynamics.

The negative benthic effects (i.e., the lower diversity of macrofauna and meiofauna found in the impact zone) noted here are assumed to result from toxicity of contaminants associated with the oil spill. It is proposed here that the lower diversity is a direct result of loss of species that are more sensitive to the presence of the toxic compounds. A good indicator of toxic effects is the sum of the PAH compounds [26] and sediment quality guidelines have been developed for shallower estuarine and marine benthic fauna [27, 28]. The concentrations of PAH related to responses reported here are consistent with the lower thresholds for responses reported in shallow water, but are half the corresponding concentrations for upper thresholds, indicating deep-sea fauna may be more sensitive than shallow fauna [29].

## References

[1] Kerr RA. A new kind of storm beneath the sea. Science. 1980; 208: 484–486. doi: 10.1126/science.208.4443.484 17744544 10.1126/science.208.4443.484

[2] Masson DG, Kenyon NH, Weaver PPE. Slides, debris flow, and turbidity currents In: Summerhayes CP, Thorpe SA, editors. Oceanography: an illustrated guide. London: Manson Publishing; 1996 pp. 136–151.

[3] Smith CR, Kukert H, Wheatcroft RA, Jumars PA, Deming JW. Vent fauna on whale remains. Nature. 1989; 341: 27–28. doi: 10.1038/341027a0

[4] Billett DSM, Lampitt RS, Rice AL, Mantoura RFC. Seasonal sedimentation of phytoplankton to the deep-sea benthos. Nature. 1983; 302: 520–522. doi: 10.1038/302520a0

[5] Grassle JF. Slow recolonization of deep sea sediment. Nature. 1977; 265: 618–619. doi: 10.1038/265618a0

[6] Kukert H, Smith CR. Disturbance, colonization and succession in a deep-sea sediment community: artificial-mound experiments. Deep-Sea Res. 1992; 39: 1349–1371. doi: 10.1016/0198-0149(92)90073-3

[7] Haake B, Ittekkot V, Rixen T, Ramaswamy V, Nair RR, Curry WB. Seasonality and interannual variability of particle fluxes to the deep Arabian sea. Deep-Sea Res Pt I. 1993; 40: 1323–1344. doi: 10.1016/0967-0637(93)90114-I

[8] Lampitt RS. Evidence for the seasonal deposition of detritus to the deep-sea floor and its subsequent resuspension. Deep-Sea Res. 1985; 32: 885–897. doi: 10.1016/0198-0149(85)90034-2

[9] Graf G. Benthic-pelagic coupling in a deep-sea benthic community. Nature. 1989; 341: 437–439. doi: 10.1038/341437a0

[10] Aulenbach BT, Buxton HT, Battaglin WA, Coupe RH. Streamflow and nutrient fluxes of the Mississippi-Atchafalaya River Basin and subbasins for the period of record through 2005. U.S. Geological Survey Open-File Report 2007–1080. 2007. http://toxics.usgs.gov/pubs/of-2007-1080/index.html

[11] Reuscher MG, Shirley TC. Spatial and temporal patterns of benthic polychaete communities on the northern Gulf of Mexico continental slope. Hydrobiologia. 2017; 790: 233–245. doi: 10.1007/s10750-016-3034-x

[12] Timmermann A, Oberhuber J, Bacher A, Esch M, Latif M, Roeckner E. Increased El Niño frequency in a climate model forced by future greenhouse warming. Nature. 1999; 398: 694–697. doi: 10.1038/19505

[13] Roberts CM. Deep impact: the rising toll of fishing in the deep sea. Trends Ecol Evol. 2002; 17: 242–245. doi: 10.1016/S0169-5347(02)02492-8

[14] Halfar J, Fujita RM. Danger of deep-sea mining. Science. 2007; 316: 987 doi: 10.1126/science.1138289 17510349 10.1126/science.1138289

[15] Peterson CH, Kennicutt II MC, Green RH, Montagna P, Harper DE Jr, Powell EN, Roscigno PF. Ecological consequences of environmental perturbations associated with offshore hydrocarbon production: a perspective on long-term exposures in the Gulf of Mexico. Can J Fish Aquat Sci. 1996; 53: 2637–2654. doi: 10.1139/f96-220

[16] Deepwater Horizon Natural Resource Damage Assessment Trustees. Deepwater Horizon oil spill: Final Programmatic Damage Assessment and Restoration Plan and Final Programmatic Environmental Impact Statement. 2016. Available from: http://www.gulfspillrestoration.noaa.gov/restoration-planning/gulf-plan

[17] MacDonald IR, Garcia-Pineda O, Beet A, Daneshgar Asl S, Feng L, Graettinger G, French-McCay D, Holmes J, Hu C, Huffer F, Leifer I, Muller-Karger F, Solow A, Silva M, Swayze G. Natural and unnatural oil slicks in the Gulf of Mexico. J Geophys Res-Oceans. 2015; 120: 8364–8380. doi: 10.1002/2015JC011062 27774370 10.1002/2015JC011062 PMC5064732

[18] Kujawinski EB, Kido Soule MC, Valentine DL, Boysen AK, Longnecker K, Redmond MC. Fate of dispersants associated with the Deepwater Horizon oil spill. Environ Sci Technol. 2013; 45: 1298–1306. doi: 10.1021/es103838p 21265576 10.1021/es103838p

[19] Montagna PA, Baguley JG, Cooksey C, Hartwell I, Hyde LJ, Hyland JL, Kalke RD, Kracker LM, Reuscher M, Rhodes ACE. Deep-sea benthic footprint of the Deepwater Horizon blowout. PLoS One. 2013; 8: e70540 doi: 10.1371/journal.pone.0070540 23950956 10.1371/journal.pone.0070540 PMC3737147

[20] Montagna PA, Baguley JG, Cooksey C, Hyland JL. Persistent impacts to the deep soft-bottom benthos one year after the Deepwater Horizon event. Integr Environ Assess Manag. 2017; 13: 342–351. doi: 10.1002/ieam.1791 27144656 10.1002/ieam.1791

[21] Clarke KR. Non-parametric multivariate analyses of changes in community structure. Aust J Ecol. 1993; 18: 117–143. doi: 10.1111/j.1442-9993.1993.tb00438.x

[22] Clarke KR, Gorley RN, Somerfield PJ, Warwick RM. Change in marine communities: an approach to statistical analysis and interpretation, 3rd edition Plymouth: PRIMER-E; 2014.

[23] Clarke KR, Gorley RN. PRIMER v7: User Manual / Tutorial. Plymouth: PRIMER-E; 2015.

[24] Hill MO. Diversity and evenness: a unifying notation and its consequences. Ecology. 1973; 54: 427–432. doi: 10.2307/1934352

[25] Carman KR, Fleeger JW, Pomarico SM. Does historical exposure to hydrocarbon contamination alter the response of benthic communities to diesel contamination? Mar Environ Res. 2000; 49: 255–278. doi: 10.1016/S0141-1136(99)00072-0 11285729 10.1016/s0141-1136(99)00072-0

[26] Swartz RC, Schults DW, Ozretich RJ, Lamberson JO, Cole FA, DeWitt TH, Redmond MS, Ferraro SP. ∑PAH: A model to predict the toxicity of polynuclear aromatic hydrocarbon mixtures in field-collected sediments. Environ Tox Chem. 1995; 14: 1977–1987. doi: 10.1002/etc.5620141120

[27] Long ER, MacDonald DD, Smith SL, Calder FD. 1995 Incidence of adverse biological effects within ranges of chemical concentrations in marine and estuarine sediments. Environ Manag 1995; 19: 81–97. doi: 10.1007/BF02472006

[28] Long ER, Field LJ, MacDonald DD. 1998 Predicting toxicity in marine sediments with numerical sediment quality guidelines. Environ Toxicol Chem. 1998; 17: 714–727. doi: 10.1002/etc.5620170428

[29] Balthis WL, Hyland JL, Cooksey C, Montagna PA, Baguley JG, Ricker RW, Lewis C. 2017 Sediment quality benchmarks for assessing oil-related impacts to the deep-sea benthos. Integ Environ Assess Manag. doi: 10.1002/ieam.1898 28121064 10.1002/ieam.1898

